# The Cholera

**Published:** 1873-09

**Authors:** 


***The Cholera.***

Amidst the numerous conflicting statements, and diverse opinions regarding this disease, which have prevailed, during the past six or eight weeks, in the town of Lake, on the southern border of the city, and the impossibility of arriving at any definite conclusions regarding it, the JOURNAL has refrained from reporting any of the cases heretofore, in order to avoid increasing the panic which threatened more serious consequences than did the disease. To-day, August 22, in company with Sanitary Superintendent Ben. C. Miller, and Acting Inspector Simons, we made a personal and thorough inspection of the "infected district," lying south of thirty-ninth street, west of State and east of Clark, and altogether outside of the city limits, and in the town of Lake. The cholera hospital was first visited, and was found in excellent order, under the care of Mr. Rofe, the newly appointed interne. There were found here three convalescents presenting no symptoms sufficiently characteristic to indicate the pre-existence of any special malady. In a number of private residences visited, the patients had either died or recovered, but two convalescents were found in the same negative condition. At No. 971 Butterfield Street, a German child of four and a half years had just died after an illness of twelve hours. The evidence furnished by inspection of the corpse a few minutes after death indicated clearly that the cause of death had been cholera. In the same house a child of about three years had diarrhœa.

The district is apparently in good sanitary condition, the surface of the ground dry, and there is no stagnant water to be found. The houses are generally of two stories, of wood, and new, the inhabitants principally German. The water supply has hitherto been bad, having been obtained from what are called by courtesy "wells," one of which when measured was found to be two feet deep and three feet from the surface of the ground; these have been rendered unavailable by the Board of Health, and the Board of Public Works has provided a supply of pure lake water, with the necessary hydrants. There is no necessity whatever for alarm, and unless one should use some diligence and have unusual facilities, as in our own case, for the detection of the disease, it would escape notice altogether.

The measures taken by Dr. Miller and Dr. Simons, for the detection and arrest of the disease in its preliminary stages, have been very effectual, and the prospect is now, that with continued efforts in the same direction, the few remaining germs of the disease will be eradicated. H.

At the last special meeting of the Chicago Society of Physicians and Surgeons, held on Monday, 18th of August, certain irregularities were perceptible in the proceedings which merit especial attention from the officers and members, in order that their recurrence may be prevented.

Of these irregularities two were especially prominent; first, the publication of the proceedings by the reporters of the secular press, not having been previously revised and corrected by the regular reporter of the Society, whereby many errors and misunderstandings might have been suppressed; and secondly, the participation in the proceedings by persons not members of the Society, without invitation of, or introduction by, the presiding officer, some of whom are notoriously disreputable, and whose names alone published in connection with the Society will be sufficient to constitute a stain upon its hitherto stainless record.

We hope that, in future, when the proceedings shall be deemed to possess sufficient interest to the general public, to excuse their publication in the secular press, they may be reduced to writing by the regular reporter of the Society, and transmitted through that channel; and further we trust, that, should the meetings of the Society again be intruded upon and its discussions interrupted by unauthorized and uninvited guests, the presiding officer will promptly silence all such, in the most summary manner. It is time that these impudent pariahs of the profession be taught their true place, and that is, outside the pale of respectable association. H.

